# From Kiyoshi Shiga to Present-Day Shigella Vaccines: A Historical Narrative Review

**DOI:** 10.3390/vaccines10050645

**Published:** 2022-04-20

**Authors:** Crystal M. Herrera, Jessicia S. Schmitt, Erum I. Chowdhry, Mark S. Riddle

**Affiliations:** Reno School of Medicine, University of Nevada, Reno, NV 89557, USA; crystalherrera@med.unr.edu (C.M.H.); jessiciaschmitt@med.unr.edu (J.S.S.); echowdhry@med.unr.edu (E.I.C.)

**Keywords:** bacterial diarrhea, history of medicine, shigella, vaccinology

## Abstract

We are at an exciting moment in time with the advancement of many vaccines, including a shigella vaccine for the world. It is instructive to look at the long road that some vaccines have traveled to recognize the remarkable accomplishments of those who were pioneers, appreciate the evolution of scientific and applied technology, and inform the future history of a vaccine that would have great potential for global health. To achieve this valuable retrospective, a narrative historical literature review was undertaken utilizing PubMed and Embase databases with relevant search terms. Retrieved articles were reviewed and information was organized into historical themes, landmark discoveries, and important vaccine development parallels. The literature reviewed was synthesized into major eras of shigella vaccine development from pathogen discovery and first attempts to empirical approaches of killed whole-cell and live-attenuated approaches, and a modern era that applied recombinant DNA engineering and structural vaccinology. The history of shigella vaccine development has largely followed the evolutionary path of vaccine development over the last 120 years, but with important lessons learned that should be considered as we embark on the future chapters of bringing to the world a safe and effective vaccine for global health.

## 1. Introduction

At present, there are no less than 10 vaccines that have entered clinical development with at least 2 that are in phase II clinical trials in Africa (ClinicalTrials.gov Identifiers: NCT04602975, NCT04056117), one with evidence of proof of efficacy in a controlled human infection challenge model, and a well-considered and feasible clinical development pathway forward to licensure [1]. While it is exciting to be at this juncture, it should also be recognized that it has been 125 years since the discovery of the bacteria that we now call shigella which has taken countless lives over the centuries and is estimated to globally cause 188 million cases of shigellosis, and 164,000 excess deaths annually [2]. The story of why it has taken so long is important, even if unanswerable. A reflective review of the history of shigella vaccine development is important to both appreciate and guide us into the future. As we know, vaccine development is the first challenging step towards a vaccination solution for an infectious disease, where an equally challenging step is to get such a vaccine utilized by those who need it the most. Thus, to celebrate and recount the past, as well as envision and prepare for the future, we conducted a narrative historical review of shigella vaccine development from discovery to present day.

## 2. Methods

**Figure 1:**
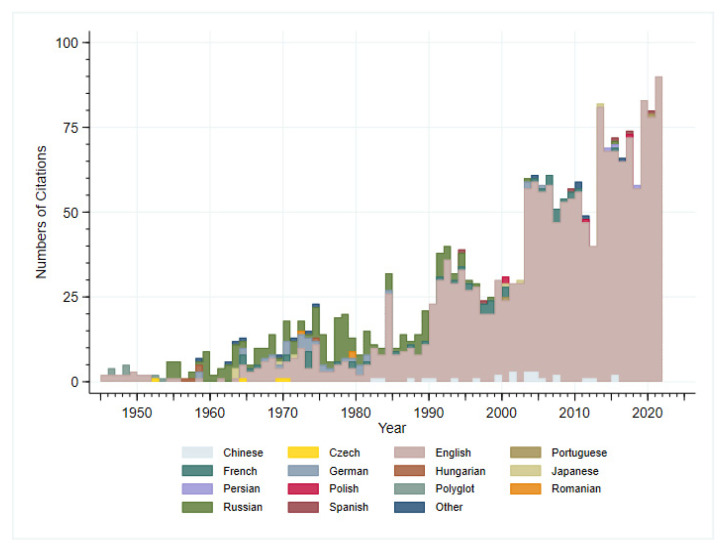
Number of citations in Embase on shigella vaccines by year and language of article (N = 2078).

For this narrative literature review, we searched scholarly databases including PubMed and Embase. Search terms included “shigell*” and “vaccine” to identify an initial set of articles to read and review. Titles and abstracts from all articles from these databases were retrieved. Articles which described the development and testing of shigella vaccines were retrieved. Reference lists of these cited articles were also reviewed and attempts to obtain articles relevant to the history of shigella vaccines were sought. Articles in non-English language were excluded (although English language abstracts were read and included if relevant information was available). The goal of this narrative review was to collect and summarize quantitative and qualitative information about the historical aspects of shigella vaccine development from time of initial discovery to present using archival research methods to construct an historical account on vaccine milestones and major discoveries of importance to vaccine development. Given the nature of these historical research methods, additional topics were searched via Google and other search engines as they related to foundational changes in the understanding of shigella which had influence on vaccine development. Given the more than 120-year history, it is very difficult to detail all the important discoveries and applications related to shigella vaccine discovery. Over the past 12 decades of research on shigella and shigella vaccines, there have been multiple comprehensive review articles written which include specific topics such as pathogen discovery, microbiology, pathogenesis, animal models, and vaccine development. To maintain an overarching historical narrative yet strive to be complete in covering all the important historical discovery, we have drawn important interpretations from these reviews and explicitly called out these works that the interested reader of history might seek out. Indeed, the body of scientific reporting on shigella vaccines is immense. An initial Embase search with the terms shigell* AND (‘vaccine’/exp OR vaccine) yielded 2078 results dating from 1945 to 2021 (although we know of and have included references that go back to the time of shigella discovery) (Figure 1). Furthermore, shigella vaccine development has been an international effort and regrettably we were not able to review non-English language articles, although we have included references of key papers in this account. Finally, given the historical research methods employed, we fully recognize content analysis limitations, and potential bias in interpretation, description, and interpretation of historical sources.

## 3. Results

### 3.1. Discovery and the First Vaccine Attempt

**Figure 2:**
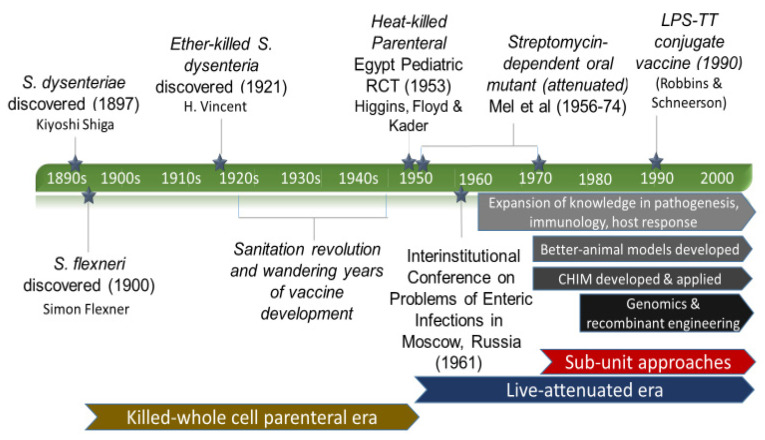
Shigella Vaccine Development History—major achievements and influences (LPS-lipopolysaccharide, TT-tetanus toxoid).

Dysentery comes from the medieval Latin dysenteria, from Greek dysenteria to mean dsy- “bad, abnormal, difficult” + entera “intestine, bowels” and was first coined by Hippocrates and was meant to cover a broad syndrome of bowel abnormalities [3]. Only in the last couple of centuries did the specificity of the word come to mean an illness characterized by bloody diarrhea attributed to amebic and bacterial causes which were also commonly referred to as ‘bloody flux’ in the 18th and 19th centuries [3]. It was in this golden age of microbiology in which standardized microbiological techniques were developed and in which most of the disease-causing bacteria were discovered, and the predominant bacterial cause of dysentery, shigellosis was named. An excellent account of the discovery of Shigella by Lampel et al. was published a few years ago and provides a definitive review of its discovery and naming [3]. A brief recounting is worth describing and reminds us as with many discoveries of this time where information was not readily accessible around the world, antecedent and convergent descriptions of this bacillus were described by other bacteriologists including Andre Chantemesse and Fernand Widel, but credited to the microbiologist, Kiyoshi Shiga, who was able to culture the bacteria for the first time from a child of an 1897 epidemic that killed over 22,000 people with a noted case fatality rate of 25% [4]. At the time of the outbreak, it was referred to as a *sekiri* outbreak, which translates to “red diarrhea”. For Shiga, the description of this bacteria which caused devastating disease, particularly among children, was impactful and drove him to immediately try to address this problem which afflicted so many. Thus, in addition to the discovery of the bacteria, Dr. Shiga was also the first to attempt to develop a vaccine against this scourge. In 1899, just two years after Dr. Shiga described the bacterium, he created a heat-killed whole-cell vaccine to which he injected himself. He reported that the local reaction was so severe that it required incision and drainage [5]. Dr. Shiga then went on to develop a serum-based passive immunization and an oral vaccine, which was used in Japan with observational (non-controlled) studies published in German- and Japanese-language journals [5]. In later years, Dr. Shiga was reserved in remarks about the efficacy of vaccines and the paramount importance of public health practices. His reflections on this time are best captured in his prescient remarks in 1936 where he stated, “...I may say that practical application is more difficult to attain than the search for the causes of disease.” —a sentiment that would hold true for decades to come in the history of shigella vaccine development [5]. The journey of the development of a shigella vaccine begins (Figure 2).

### 3.2. Eras of Development

#### 3.2.1. The Empirical Vaccine Era

While Dr. Shiga may have abandoned vaccine development, others did not, and attempts to replicate the success of many vaccines of the day commenced with killed whole-cell and live-attenuated strategies (many of which continue to be pursued in the modern vaccine approach era). The empirical approach era was characterized by strategies that involved growing up the pathogen, isolating it, and then either killing it or weakening it prior to injection [6]. This approach saw the development of many successful (and equal number unsuccessful) vaccines including rabies, typhoid, yellow fever, diphtheria, pertussis, and influenza to name a few. 

Subsequent attempts of this era focused on the development of killed-whole cell anti-dysenteric vaccines by various methods. Alexandre Besredka, a French biologist and immunologist, immunized mice with sensitized vaccines, Tamezo Kabeshima, a researcher in Shiga’s laboratory who visited the Pasteur Institute, immunized rabbits via injections of dead bacilli that were killed via the method of d’Herelle, a bacteriophage, and others attempted combinations of *S. dysenteriae* with microbes such as *Vibrio cholerae* and *S. Typhi* (referred to at the time as *B. cholera* and *B. typhoid)* [7]. These attempts all faced the same dilemma of frequent painful reactions, which greatly hindered the pursuit of the whole killed cell approach [7].

Jean Hyacinthe Henri Vincent, a French physician and associate professor at Val-de-Grace, and Inspector of Hygiene and Epidemiology in the French Army, noted the continued burden of shigella in the military, as well as the prior attempts and reactogenicity challenges using different methods of heat-killed vaccines and came to the problem with a different approach. In the French army laboratory of that time, Vincent pioneered the development of typhoid and paratyphoid vaccines which were developed in 1910, permitted by the Académie Nationale de Médecine in 1911, and made compulsory by the French Army in 1915 [8]. This vaccine, as well as the Pastuer Institute’s heat inactivated vaccines, were credited with the substantial reduction in mortality after vaccine program introduction. The technique used for the Vincent typhoid vaccine was through a technique where young cultures were sterilized by exposure to ether. Given the apparent safety and effectiveness of these vaccines, Vincent and the Army laboratory started applying this method to other enteric pathogens such as cholera and shigella. Vincent’s only paper on this shigella vaccine approach was published in 1921, where he described the construct and testing of a polyvalent vaccine that contained five strains of Shiga type, one Strong type, two Flexner type, and four type Y; the cultures of these strains were sterilized by ether [7]. These initial vaccine designs were advanced into rabbit studies where complete immunity was conferred in a virulent infection model (not further described). Having this animal evidence (no toxicology studies described), Vincent proceeded to launch human testing with a stated goal of developing a vaccine with the “least possible reaction.” What is described as a first step is a phase 1/2 study where a small dose (250 million bacteria) was given to 11 men in a French prisoner of war camp (Box 1).

Box 1 **A solemn remembrance of the inhumanity of past research.**

It is important to pause here and remark upon this abhorrent reminder of the cruelty in clinical research that took place in decades past. We know the world was in conflict and researchers seized the opportunity to use prisoners of war to progress the current understanding of disease states and vaccines. While the inhumanities observed during World War II are a stark reminder of the inhumanities that have happened and which led to the world mobilized response leading to the Nuremberg Code, we also solemnly recount this observation from World War I did not result in a global mobilization to stop such actions. In the Vincent study, there was no description of consent being obtained for the prisoners, however it was noted that patients (prisoners) who were hospitalized were provided antidysenteric serum and medical treatment [7].

After the initial study of the 11 subjects where no reactions were noted, a series of dose escalation studies of 500 and 750 million bacilli were given to groups of 600 and 1575 prisoners, respectively. Reactions observed were mild and noted to be less numerous and less severe compared to the paratyphoid and typhoid vaccines that were in use. Given the enrollment population was among a camp of 3200 individuals, the researchers took the opportunity to compare the rates of shigellosis occurring among vaccinated and unvaccinated individuals. To their amazement, vaccinated subjects experienced a 12-fold reduction (89.9% vaccine effectiveness) of shigellosis compared to the non-vaccinated group, although such a design could favor a higher efficacy as the outbreak began to diminish during the study period, and thus true effects are difficult to know. While certainly tarnished, this was the first example in shigella vaccine in history where an indication of vaccine efficacy was observed against natural disease. It is unclear why, but no further development of this vaccine is described. It is known that in the early 1920s, the safety of the French typhoid vaccines was being called into question [8], combat operations had come to an end, and thus the priority of vaccine development against shigella relative to the demands of rebuilding a war-torn country may have been a factor. A side note also worth mentioning is that the ether-based bacteria inactivation fell out of favor over the proceeding years. However, currently, ether is a method that is commonly employed in development of new viral vaccines such as influenza and chikungunya [9,10].

#### 3.2.2. The Wandering Years

For the next 30 years, the historical record becomes very thin but not absent. Efforts of secondary prevention were reported in 1924, at the Serum Conference in Geneva, an antitoxin treatment for bacillary dysentery was described, and therapeutic dosage levels were experimented with favorable results for mild and moderate cases, but lack of efficacy for treatment of severe dysentery cases [11]. Concurrent with this period was the end of the sanitation revolution which aided in decreasing the caseload of Shigellosis by improving sanitation and hygiene throughout the United States and Europe [11]. Even Shiga noted ‘suppression of intestinal infectious diseases…relies upon the progress of modern public health practices’ which became feasible with the introduction of municipal chlorination, food inspection, personal hygiene, soap, disinfectants, and public health department regulations [11]. This downtrend in disease of fecal-oral transmission placed shigella vaccine research as a lesser priority during the inter-Great Wars period. However, efforts to understand the fundamental microbiology, speciation, growth requirements, pathogenesis, and immunology of the organism continued and would enable novel leaps in vaccine discovery well into the future. World War II brought about reinvigoration of the need for a shigella vaccine, as dysentery rates among allied and axis forces were high [12]. Prior to WWII, there was a 4:1 ratio of infectious disease mortality to combat mortality, with a substantial proportion of that being due to dysentery. Despite the improvements during WWII in field sanitation, vaccines, and antimicrobials, dysentery posed a significant burden, with shigellosis being responsible for about half of all dysentery cases. This perennial scourge re-launched efforts by many to develop a countermeasure. This area saw both oral and parenteral vaccine approaches which were met with mixed results (Supplementary Information Table S1). This period was characterized by a variety of monovalent and polyvalent killed whole-cell approaches that were administered orally or parenterally, most often in high-risk populations during seasonal outbreaks and in non-randomized designs, although some with control groups. The challenge of design and observational nature of many of these reports, some of which suggested protection, failed to generate compelling evidence due to concurrent quarantine and isolation activities often being used confounding the attribution of vaccine effects. Discouragingly, in studies where robust control populations were used such as the trial conducted by the American-Egyptian team of Higgins, Floyd, and Kader in Egypt in 1953, killed whole-cell vaccines failed to protect [13]. 

#### 3.2.3. Live-Attenuated Vaccines Emerge

It would appear in 1961 that a new day in shigella vaccine development would dawn. The challenge of shigella vaccine development was discussed and deliberated at the Interinstitutional Conference on Problems of Enteric Infections held in the spring in Moscow, Russia and one Yugoslavian Army Colonel David M. Mel was in attendance [14]. The failures of the prior decades of attempts at developing vaccines were recounted, and fundamental challenges including not even fully understanding the mechanisms of immunity (still present today) were belabored. A concluding recommendation from this meeting was that new approaches were needed, and new principles adopted for the development of vaccines against shigella. 

In a clear and incremental six-part manuscript series published in the Bulletin of the World Health Organization between 1965 and 1971, Mel and colleagues described a tremendous body of work from vaccine discovery to fundamental immunology, and incremental advancement to well-designed field trials to fully understand important regimen factors such as schedule and doses on vaccine efficacy in both children and adults [15]. In a learned but undifferentiated first step, Mel and team generated 14 different vaccine constructs for *S. flexneri* 1 which were either detoxified or prepared in a manner in order to increase immunogenicity (adjuvanted) for parenteral administration and intracerebral challenge in mice. In addition, he developed five vaccine constructs from *S. flexneri* 2a which were prepared for parenteral or oral administration to prevent an oral challenge in mice that was recently described by Freter [16]. The main conclusions from this work were that parenteral administration of vaccines (some but not all) while protecting against intracerebral challenge, did nothing to prevent enteric colonization in mice. It was only when vaccines that were administered by the oral route and consisting of living cells that resistance to colonization was seen. It appears that this was the fundamental evidence that Mel and colleagues needed to establish new principals and think about shigella vaccine development. Fundamentally it was observed that intestinal mucosal antibodies and not serum antibodies were an important factor in protection against infection, and parenteral administration was not the route that was going to induce strong mucosal responses. The observation that only living cells administered by the oral route conferred protection in the mouse experimental model, was the finding that launched the remainder of work focusing on oral live-attenuated vaccines, an effort though with much more sophistication, continues to be pursued today.

A full recounting of the series of published studies by Mel and colleagues advancing knowledge around the live-attenuated oral vaccine approach would be a most interesting and lengthy endeavor which the avid reader is encouraged to pursue [14,15,17,18,19,20,21,22,23,24]. However, the important and foundational aspects of this work are briefly described here for historical reflection. While the notion of a live oral vaccine was essential, it was also recognized that attenuation of the oral infection was needed so as to be safe. Mel originally did this by taking *S. flexneri* 2a strain 2457T (obtained from the Walter Reed Army Institute of Research (WRAIR)) and plating it on heavy suspensions of nutrient agar plates containing 400 ug/mL of streptomycin and selecting mutants which grew. The thought was that these weakened bacteria would not multiply but would induce the necessary immune responses “if given repeatedly in adequate doses.” It is not exactly clear, but this description of developing an attenuated vaccine for human use using the streptomycin-dependent selection technique may be the first. The idea may have come from Herzberg and Elberg who were working on a brucellosis vaccine for animals in the early 1950s [25], although streptomycin-dependent bacterial selection and characterization was also going on with human pathogens of *M. tuberculosis* [26] and *Staphylococcus aureus* [27] in the mid-century. Of interest, this method is still being used today in development of animal vaccines. Other important experiments were studies demonstrating no evidence of reversion (e.g., growth without streptomycin), and subsequent incremental advancement of oral vaccination in human volunteers beginning with low doses and numbers of subjects, and incrementally testing muti-valency admixtures, with both safety and immunogenicity (phagocytic activity) readouts to look at both homologous and heterologous responses [19]. The experiments noted that the duration of vaccine shedding (bacterial strain recovery in the stools) was directly related to inoculum dose, and use of pre-vaccination sodium bicarbonate also increased vaccine shedding. Upon optimizing safety, dose, and regimen, Mel and colleagues advanced to field trials among military service members where they employed a monovalent five-dose (1, 4, 7, 10, 13 day) *S. flexneri* 2a vaccine given to 355 soldiers in six units and compared them to 382 unvaccinated soldiers in the same units (non-randomized and unequally distributed), and assessed outcomes of *S. flexneri* 2a carriage, *S. flexneri* 2a associated dysentery, and non-*S. flexneri* 2a dysentery. Remarkably, across all six units, there were no cases of *S. flexneri* 2a dysentery in the vaccinated soldiers, whereas there were 21 *S. flexneri* cases in the unvaccinated controls. No effect on colonization was noted and there was a reduction in non-*S. flexneri* 2a dysentery (vaccinated 10.4%, unvaccinated 16.5%). It was also noted in this study that a trivalent vaccine given at a lower CFUs and in a three-dose series (1, 4, 7 days) did not confer protection. Follow-on studies within military personnel were carried out with bivalent vaccines utilizing the five doses (every 3rd day increasing inoculum) in a similar non-randomized but well-controlled field study demonstrating slightly less, but still high levels of protection (~85%) against infection by both subtypes contained in the vaccine [15]. Following these studies, lyophilization was introduced and demonstrated that efficacy was not reduced which allowed the promise to improve scalable production, and efficacy was demonstrated in pediatric populations although not without challenges of reactogenicity, the requirement for bicarbonate pre-dosing and at least four doses, as well as relatively short-term protection (6–12 months) [15]. While ultimately concerns about reversion, lack of understanding the exact mechanism of attenuation, and challenges in large-scale manufacturing prevented further advancement, these remarkable series of studies were a testament to an extraordinary achievement in shigella vaccinology and ushered in an era of shigella live-attenuated vaccine development. The reader interested in learning more about this era of live-attenuated vaccines that is actively continued to this day is directed to a very well written scholarly review by Levine and colleagues that outline the advances and set-backs of this strategy [28]. A strategy that has been pursued by multiple nations including Russian, Romanian, Polish, China, and multiple US research teams that has led to highly characterized attenuated strains and continue to bring hope, although strong evidence for efficacy in adults has remained elusive, and the formidable challenge of the child’s intestine and environmental enteropathy has proven to be a major hurdle in induction of both vaccine take and robust immune responses not just for shigella vaccines, but for other oral attenuated vaccine such as rotavirus and cholera [29]. 

#### 3.2.4. On the Origins of the Shigella Controlled Human Infection Model

During this era of empirical vaccine development, the first controlled human infection model (CHIM) to test efficacy of a shigella vaccine was used by Shaughnessy and colleagues [30] reported in JAMA in 1946, although this was not the first time a human challenge model was used for vaccine evaluation [31]. Following pre-clinical studies in animals and assurance of susceptibility to antibiotics, the research team selected a series of S. *flexneri* strains to be used in a series of experiments design to determine the minimum infective dose for man, as well as evaluate the efficacy of two vaccine constructures including polyvalent vaccines containing irradiated and heat-killed antigens. The testing was conducted among volunteers in an isolation ward at the Joliet Penitentiary in Illinois. Dose identification was conducted in groups of four volunteers and varied strain, dose, and use of milk as a vehicle, coadministration with bicarb and other medication coadministration techniques. The research team also described an unconventional coadministration of cultured organisms combined with encapsulated feces from health controls to serve as an “adjuvant” to the challenge as early attempts at getting consistent attack rate failed. Ultimately, they were only able to achieve an attack rate greater than 50% when they gave a combination of 625 million organisms of four different strains where two out of three challenged volunteers developed a moderate form of clinical dysentery. This regimen was not repeated, nor did they attempt any further combination of strains. In total, 39 volunteers were used to identify the combination challenge for the follow-on vaccination experiments. In a subsequent series of experiments 25 volunteers receiving a parenteral heat-killed vaccine, 28 volunteers receiving a parenteral irradiated vaccine, and 30 controls were challenged with the four-strain mixture. Two doses of vaccines were administered two weeks apart and volunteers were challenged two-weeks after the last dose. Disappointingly, 72%, 82%, and 63% developed dysentery demonstrating no vaccine effect. There were varying attack rates and severity of illness across multiple cohorts which may have confounded the ability to discern an effect. Much was learned from the detailed observations of shigellosis under these controlled conditions, the clinical effectiveness of antibiotic therapy as one, as well as antibiotic treatment in the reduction of the carrier state.

#### 3.2.5. The Modern Approach Era

In addition to the work of Mel and others on whole-cell approaches during the 1960s, this was also a time of substantial advances in our understanding of pathogenesis of the varied species of shigella, immunology, as well as molecular and synthetic biology. The breakthroughs in shigella pathogenesis knowledge during this time is described in an excellent 1983 review by Mike Levine et al. [32]. Accompanying this time of fundamental understanding of biology and vaccinology, several branches of vaccine development saw promising starts but eventually stalled or were overtaken by other strategies. For example, *S. dysenteria* 1 and its associated toxin which caused global outbreaks of severe diarrhea in the 1970s–1990s was a major focus of seminal work by Keusch and colleagues [33], although it was described 50 years earlier by Conradi, Neisser, and Shiga himself [34]. Despite the success of diphtheria and pertussis toxoid vaccines, shigella toxoid vaccines were developed and induced very high levels of circulating antibodies but did not ameliorate disease in a non-human primate disease model [32], suggesting that systemic anti-toxin antibodies were not enough to counteract toxin-mediated effects. Additionally, in the early 1960s, the team of Anne and Guy Youmans began an approach for a vaccine against *M. tuberculosis* which utilized a cellular rupture and ultracentrifugation methods to obtain the ribosomal fraction and inspired a new approach to vaccine development [35]. Russian researchers led by Vadim Levenson from the Gabrichevsky Moscow Research Institute of Epidemiology and Microbiology applied this approach to shigella in the 1970s through the 1990s [36,37]. In a series of studies, they were able to demonstrate efficient manufacturing and efficacy in animal models. While the composition of the ribosomal vaccine preparations varied, experiments would suggest that the polysaccharide nature of the O-antigen was most important, however the ribosomal constituents were important for increasing the immunogenicity to O-antigen. Ultimately, this approach dwindled in the mid-1990s, although other more refined sub-unit approaches would emerge and reinforce the emerging effort of polysaccharide-protein conjugate vaccines which would soon take hold.

From the Sam Formal lab of the WRAIR in the 1960s, two fundamental discoveries were made that would change the course of shigella vaccine development for the next few decades. In a landmark paper, Eugene Labrec and colleagues set out to understand what the cause of the ulcerative lesions was that was seen in the intestinal tract in human shigellosis, which at the time was hypothesized to be mediated by excreted toxins of colonizing bacteria [38,39]. While at the time small animal models such as rabbits, mice, and guinea pigs were in use, none of them recapitulated human disease. Furthermore, the recent description of a “smooth” variant of *S. flexneri* 2a (2457O) that was found to not cause any disease in an intraperitoneal injection guinea pig model gave them the opportunity to conduct a comparative study of a tissue culture invasion assay (HeLa cells), intraperitoneal injection model in guinea pigs with a recently developed non-human primate rhesus macaque model and advanced histopathology. Remarkably, they were able to discriminate the virulent (2457T) and avirulent (2457O) strains in each experimental model and bacteria of the 2457T was only found to invade the HeLa cells. Furthermore, in the non-human primate diarrhea was found and the typical ulcerative lesions and in frozen section of the tissue, fluorescent anti-*S. flexneri* 2a antibody was found in the lamina propria along with inflammatory and epithelial cells. It was these observations that set the community to try and fundamentally understand epithelial cell invasion. Other fundamental discoveries that came out of the Formal lab leveraged emerging methods of genetic engineering to create hybrid strains from conjugation experiments between an innocuous bacterial strain *E. coli* K-12 and virulent *S. flexneri* 2a 2457T [18]. One graduate student named Stanley Falkow demonstrated that replacement of the shigella chromosomal region between the Rha+ and Xyl+ genes with those of *E. coli* K-12 could remove virulence [39]. However, the research teams could not convert the *E. coli* K-12 into a virulent strain through these methods. It took the complementary efforts by Dennis Kopecko in the Formal Laboratory and Phlippe Sansonetti at the Institut Pasteur working with *S. sonnei* to discover virulence determination was due to the loss of a large plasmid [4,40], which was ultimately identified in *S. flexneri* as well [41]. Interestingly, the original observation by Falkow would end up being explained by an important chromosomal gene VirF of *S. flexneri* that serves as an important regulatory of the virulence gene on the plasmid [42]. As might be expected, the identification of a virulence plasmid had ripple effects in the community with multiple groups setting out to better understand the genes and proteins that were involved in virulence [43]. Finally, with the remarkable advancement of understanding of pathogenesis in the basic sciences, also came advances on the clinical side. Firstly, 1969 saw the first human challenge model by Dupont and colleagues [44], which was used three years later to demonstrate that natural infection could induce protection against rechallenge [43] which set the bar for future vaccine strategies. 

Emerging from the understanding of shigella genetics came the 1990s and learned attempts to use this information to develop new vaccines including the insertion of *S. sonnei* O-antigen genes into Ty21a *Salmonella Tyhpi* live-attenuated vaccine under development [45], and other vectored live attenuated approaches including an E. coli K-12 vectored approaches [46,47], all of which failed. Live-attenuated approaches with rational site directed mutations emerged and for the first time in 1999, a live-attenuated vaccine co-developed by the US Army and the Institute Pasteur demonstrated strong immunogenicity and protection in a CHIM, although it proved to be too reactogenic in US adults [48], but not immunogenic in Bangladeshi adults and school children [49]. Researchers out of the University of Maryland also leveraged this new understanding of genetics and virulence to develop other rational attenuated strategies which continue to be advanced [50]. While not rationally designed, the T32-Israti strain developed by the Romanians in 1984 saw substantial development in Romania and China, though it was never commercialized [51]. The live-attenuated approach whether by targeted gene deletion or using other bacteria to vector important epitopes continues to present. While none has yet to demonstrate both efficacy and safety, research teams continue to try and thread the needle of a vaccine that will induce immunity, but not too much reactogenicity. Also of growing challenge is the recognition that a vaccine that works for the adult in a high-income country (HIC), may not work as well in the child of a lower-middle income country (LMIC) where changes to intestinal response to vaccines appears very different. Mike Levine and colleagues provide a well detailed and expansive review on the continued challenges and opportunities for live-attenuated and vectored shigella vaccines [28].

In addition to rationally designed live-attenuated approaches, smart sub-unit vaccines approaches also took root with the O-antigen as its target. These started in the 1990s by John Robbins and Rachel Schneerson and colleagues [52] who had been working on polysaccharide conjugate vaccines for typhoid, pneumococcus, and *Haemophilus influenzae* type b since the mid-1980s. Although the enhancement in immunogenicity when polysaccharide antigens were covalently linked to proteins was first discovered by Avery and Goebel in 1929 [53], it was more than 50 years later that this would start being used in development of vaccines. The research team at the National Institute of Child Health and Human Development (NICHHD)of the NIH credit the pioneering work of Maxwell Finland on capsular polysaccharides and their relationship to infectious diseases, the similarities in pathology between non-typhoidal salmonella and shigella, and the strong evidence supporting the invasiveness of shigella due to their O antigens and evidence of serotype specific protection in humans correlating with serum anti-O antigen antibodies [52,54]. Following a Phase I study among adults in the US Army by David Taylor and colleagues [55], a bi-valent conjugate vaccine (*S. sonnei* and *S. flexneri* 2a bound to Pseudomonas aeruginosa recombinant exoprotein A) was advanced into a series of studies in the Israeli Defense Force and demonstration of efficacy in a field trial [56,57]. These results spurred advancement of this vaccine into a phase 3 evaluation of a bivalent vaccine in an age descending design in Israeli children [58]. While encouraging, the results were not completely satisfying as the efficacy in the 3–4-year-old group was approximately 71%, but substantially lower in the 1–2-year-old age-group which was the target population of interest. While this construct was not further advanced, it spurred the development of newer conjugate vaccines based on biological (instead of chemical) conjugation methods [59,60], and synthetic carbohydrate chemistry [61]. These approaches to ‘build a better mousetrap’ through contemporary application of bioengineering and synthetic vaccine technologies are currently in field trials in Africa and show much promise and hope (ClinicalTrials.gov Identifiers: NCT04602975, NCT04056117).

While the conjugate vaccines may be most promising, there is still uncertainty as to whether they can protect in the youngest age-groups. The shigella vaccine development community is a dogged one and out of the important advances in pathogenesis previously described have also emerged unique and novel smart vaccine design strategies. For example, researchers at the WRAIR led by Edwin Oaks, pioneered the development of a sub-unit complex vaccine based on the Type III secretion system apparatus that continues to advance and be refined [62,63]. In another novel strategy, genetic engineering was used to create well defined and characterized out-membrane molecules that contain defined antigenic and costimulatory components called generalized modules for membrane antigens (GMMA) using reverse vaccinology principles [64]. A major truth in vaccine development is that the more shots on goal the better. Certainly, at this moment in history there are multiple promising approaches.

### 3.3. A Glimpse into the Future

It was Walter Orenstein who said it best, “Vaccines don’t save lives. Vaccinations save lives.” [65]. While the current arc of history is still in the phase of creating a vaccine that will work, we must consider that that is only half of the story. If a shigella vaccine were licensed and globally available tomorrow it would still face many challenges. Another aphorism that is appropriate at this juncture is from Mark Twain who is claimed to have said, “History may not repeat itself, but it does rhyme a lot.” It is thus with this in mind that we look to the future of shigella vaccination and what we can learn from the past. Firstly, one of the most significant barriers to the utilization of a Shigella vaccines globally may be the lack of reliable information on the true disease burden. Major global health efforts such as the Global Enteric Multicenter Study (GEMS) and the Malnutrition and Enteric Disease Study (MAL-ED) have rigorously attempted to define estimates of mortality and morbidity among children under five in the developing world [66,67]. However, these estimates do not consider the long-term sequelae of shigellosis, nor the burden of disease in older children and adults which may undervalue the use of a vaccine by developers and decision makers [66]. As such, while there has been an incredible amount of effort and study on the epidemiology of shigellosis incidence and mortality, particularly among children in LMIC, more data is needed to expand our understanding of the full public health value of a vaccine which drives the political will and aids in development prioritization and ultimately vaccine availability for all important markets. We must also remember that vaccines that work well in HIC or adults, may not always work in LMIC or children. Rotavirus vaccines is one history lesson to review. It is favorable that the leading shigella vaccine candidates have entered a development path early in LMIC populations which will lead to earlier understanding of the effectiveness in these populations (ClinicalTrials.gov Identifiers: NCT04602975, NCT04056117). 

Going for a shigella vaccine is that there may be a dual market for such a vaccine to be used in travelers and the military. Such vaccines that create demand and capacity in HICs where higher prices can be charged to offset the price paid in LMIC will tend to have an advantage compared to vaccines where there are only LMIC targets. However, here again, burden of disease data is lacking and compared to ETEC or norovirus, a shigella vaccine may not be seen to have as high of demand, thus dampening industry and global health interest. Combination vaccines may also be a path forward for a shigella vaccine and the current polysaccharide-conjugate vaccines may have synergies with other pediatric conjugate vaccines recently licensed or currently in expanded programs of immunization. Lastly, there is current and growing advocacy and investment for a shigella vaccine by the World Health Organization, the Bill and Melinda Gates Foundation, Wellcome Trust, and others which brings promise when many come together for a common purpose.

## 4. Conclusions

The history of shigella vaccine development is a rich and interesting one. Like many other vaccine development efforts, it is characterized by advances and setbacks, as well as periods of robust innovation spurred by advances in pathogenesis understanding, as well as leaps of innovation that are taken from advances in other vaccine technologies. If anything, the history of shigella vaccine development has been shown to be a collective investment of many people around the globe to solve an important and tragic public health problem that claims countless lives every day. Perhaps Kiyoshi Shiga said it best during his address at the Harvard Tercentenary Conference of Arts and Sciences in Cambridge on 10 September 1935:

“The discovery of the dysentery bacillus stirred my young heart with hopes of eradicating the disease. Many thousands still suffer from this disease every year, and the light of hope that once burned so brightly has faded as a dream of a summer night. This sacred fire must not burn out.”

Those who have made so many discoveries from the past, and those who are working so diligently on the vaccines now are guided by a very bright path forward to a vaccine of tomorrow.

## Supplementary Materials

The following supporting information can be downloaded at: https://www.mdpi.com/article/10.3390/vaccines10050645/s1, Table S1: A compendium of selected clinical vaccine development efforts taking place from the 1920s to the 1950s. References [68,69,70,71,72,73,74,75] are cited in the Table S1.
