# Supplementary material for: From Kiyoshi Shiga to Present-Day Shigella Vaccines: A Historical Narrative Review
Source: Vaccines (Basel). 2022 Apr 20;10(5):645. doi: 10.3390/vaccines10050645 (PMC9145194; doi:10.3390/vaccines10050645)
Supplement: Supplementary file 1 [file vaccines-10-00645-s001.zip › vaccines-1597760-supplementary.pdf]

Supplementary Materials

Table S1. A compendium of selected clinical vaccine development efforts taking place from the 1920s to the 1950s.

Year	Author(s)	Study population	N	Study design	Vaccine construct	Primary findings	Limitations
**1924**	Wilkins and Wells [68]	Institutionalized children (Baltimore, MD)	70	Single arm introduction during ongoing outbreak with high mortality with failed isolation and quarantine measures	Heat-killed vaccine of strain obtained from outbreak. Three doses (250, 500, 100 bacilli) delivered subcutaneously every 3 days	Abrupt termination of new cases after vaccine introduction; Vaccine reactions in 2/70 (fever w/seizure in 10 mo. old & fever in 15 mo. old); local reactions were common; agglutination reactions to subset of children found in 10/11 tested	**No control group; outbreak had been running its course previously**
**1930**	Munro [69]	Children from Hillsleigh School (Colchester, England)	48	Single arm	Oral vaccine containing equal parts of Shiga's and Flexner's bacilli. 3 doses of 5 cc each delivered on 3 consecutive days	No reaction of any kind was noted. No case of dysentery occurred the patients received the vaccine 6 months ago.	**No control group; cases of diarrhea and dysentery had been occurring during the last few years**
**1938**	Paddle [70]	Patients from Caterham Hospital of the London County Council accommodating over 2,000 psychiatric patients	~2,000	Single arm with control period	Polyvalent vaccine made from cultures of *B. dysenteriae* (Flexner) of all types isolated from Caterham Hospital and other London County Council mental hospitals containing 500 million organisms per cubic cm. Administered in 2 doses of 0.5 cc, given a week apart.	No reaction noted after immunization. Both the control and inoculated period had a similar amount of primary cases and contacts (patients in a ward where a primary case occurred), yet the inoculated period had significantly less secondary cases (16 cases in inoculated vs. 46 in the control period).	**Dysenteric disease had been endemic for many years at this institution. The control population was during a different time period (1932-1936) than the inoculated population (1932-1936). In 1931, "dysentery was assuming epidemic form."**
**1943**	Caldwell and Hardwick [71]	Cases occurred in West Park Hospital, Surrey	357	Single arm	Polyvalent vaccine which incorporated the "V," "W," and "Z" strains f B. *Flexneri*. Administered subcutaneously to every patient in the ward as soon as an active case was discovered. Doses of 0.5 cc and 1.0 cc of vaccine	Short duration immunity conferred of less than 4 weeks from agglutination tests carried out. However, the vaccine was effective in reducing spread when one or two sporadic cases occurred on the ward and when patients were all vaccinated.	**No control group; an epidemic of dysentery had been occurring**

					containing 10 ^9 organisms per cubic centimeter.		
**1943**	Johns and Chalk [72]	Patients admitted to Ontario Hospital London, Ontario)	42 6	Single arm	Heat-killed oral vaccine of strain obtained from outbreak. Five doses given over 6 days. Dose on the first day was 10 cc, followed by 20 cc each day for the next 3 days. No vaccine was given on the fifth day and on the sixth day, 40 cc was delivered.	Oral vaccine was well tolerated, with no reported severe reaction. There was a significant agglutinin response following vaccination. Conferred immunity from the oral vaccine is estimated to have been effective for at least 1 year.	**No control group; bacillary dysentery was endemic**
**1945**	Klimentov a [73]	Patients of a 525-bed psychiatric hospital	59 5	Single arm with control population from a different nearby psychiatric hospital.	Polyvalent Shiga-Flexner vaccine containing two Shiga and four Flexner strains. Vaccine administered subcutaneously 3 times at six-day intervals in doses of 0.5, 1.0, and 1.5 cc containing a total of 5 billion organisms.	Overall, the vaccine was well tolerated by humans, with a total dose of nine billion bacilli. There was a significant increase in the titers of agglutinins and antitoxin noted in vaccinated individuals. The assay of the Shiga-Flexner anavaccine yielded favorable results in the subset of patients studied with no cases of dysentery occurring in the vaccinated group, compared to 29 cases of dysentery recorded in the control non-vaccinated groups of patients examined in from a nearby similar institution.	**Short period of observation following immunization of 12-15 days to check for the efficacy of immunization; small study population of 29 individuals who were part of different hospital populations; study was carried out in the midst of a dysentery epidemic and 5.2% of the study group were carriers of the Flexner microbe**

**1946**	Shaughnessy [30]	Volunteers in an isolation ward of the hospital at Old Prison, Joliet Penitentiary (Joliet, Illinois)	122	Randomized Control Trial	Irradiated and heat-killed polyvalent vaccines from isolates collected at Illinois Department of Public Health Laboratories. 3 doses of 2.5 cc of vaccine containing 4.8 billion organisms per cubic cm, spaced 2 weeks apart. The challenge with ingestion of live Shigella organisms was completed 2 weeks after the final vaccination	18/25 developed dysentery in the heat killed vaccine group. 23/28 developed dysentery in the irradiated vaccine group. 19/30 developed dysentery in the control group. Local reactions tended to be more severe in the heat killed vaccine group compared to the irradiated vaccine group, although reactions were mild to moderate. Thus, parenterally administered vaccine proved to be ineffective in the prevention of naturally occurring Shigella infection, although a high degree of immunity was generated in mice models and there was also a significant increase in mouse protective antibodies in human subjects.	**Variations from experiment to experiment were significant, even in the control group with the incidence of dysentery that there appeared to be no significant difference between any of the groups.**
**1948**	Hardy, Decapito & Halbert [74]	Patients from a New York State Institution and from an Illinois institution, both for psychiatric patients	921	Randomized Control Trial	Vaccines were prepared from the same strains used by Shaughnessy and associates to constitute a six-organism polyvalent vaccine. The three different processing techniques included vaccines prepared by killing organisms with heat, formalin, and ultraviolet radiation. Adults were administered 6.25 billion organisms in 3 doses (1.25, 2.5, and 2.5 respectively) at weekly intervals for all vaccines except those containing the modified Shiga toxin. The Shiga containing vaccine contained 1.5 billion organisms and was administered in 3 equal doses. Children were administered	The vaccines caused "wide erythematous reactions with relatively little edema or soreness." The addition of modified Shiga toxin or Shiga organisms resulted in severe reactions (even when the vaccine dosage was reduced to 1 billion organisms). Severe reactions to the dosage of 1 billion organisms were also recorded for vaccines containing organisms killed by irradiation, heat, and formalin. The ultraviolet preparation was tolerated relatively better than the others. Infants and children were noted to have severe reactions even when they were given significantly reduced dosages. At the New York institution, 140/265 individuals (53%) in the control group contracted Shigella infection and 135/265 individuals (51%) in the vaccinated group became infected. At the Illinois institution, 71/134 individuals (53%) in the control group and 133/259 individuals (51%) in the vaccinated group became infected. Thus, the parenterally delivered vaccines had no significant value in the control of Shigella infections. The response to booster inoculation 6-8 months after the initial vaccination was also	**Patients in these institutions already had a high baseline endemic incidence of Shigella infections. There was high transmission of disease in the setting of failed quarantine measures.**

					one-half to one-fourth of the adult vaccine doses, depending on their age and weight.	looked at. Results suggested that there was no significant improvement in immune response in comparison to solely completing the first series of vaccination.	
**1948**	Thale and Opper [75]	Patients who resided in the following 3 wards: elderly, disturbed, and deteriorated women	428	Randomized Control Trial	Polyvalent vaccine which incorporated the "V," "W," and "Z" strains of B. *Flexneri*. 3 doses delivered at weekly intervals. Injections of 0.5, 1.0, and 1.0 mL of the vaccine containing 500 million organisms per ml was delivered subcutaneously.	The incidence of infection in the vaccinated group was slightly higher than in the control group, although the difference was not statistically significant. Age and general health did not show a significant difference in morbidity. The lack of previous exposure to infection as well as unclean personal habits were factors associated with increased morbidity.	**Poor hygiene amongst the mentally ill patients contributed to a higher rate of transmission and cases of diarrheal disease. All patients lived and ate together, with no attempt to segregate the various groups. There was no placebo injection given to the control group.**
**1955**	Higgins, Floyd & Kader [13]	Egyptian village children	200	Randomized Control Trial	Monovalent Shig. Flexneri 3 heat-killed vaccine. Three doses delivered subcutaneously at weekly intervals in the divided amounts of 0.3 mL, 0.3 mL, and 0.4 mL. The vaccine contained 2.5 billion organisms per mL.	Vaccines tolerated in all subjects with minimal side effects and no severe reactions. After a six month observation period following vaccination: 25/93 children who received the vaccine were infected with Shig. Flexneri 3 organisms compared to 18/88 from the control group. The number of infections from other Shigella organisms and the number of episodes of diarrheal disease were approximately the same in the control and study group. Thus, the Shigella flexneri 3 vaccine was not effective in preventing infection from Shigella flexneri 3 or other associated diarrheal disease in the group of Egyptian village	**At the end of the 6 month observation period, there remained 88/100 in the control and 93/100 in the vaccinated group. This was due to: attrition from deaths, non-cooperation, or changes in residence. Some deaths in the control (5/100) and vaccinated group (3/100) were due to diarrheal disease of unknown etiology. The control group was not given a placebo inoculation.**

	children who lived in a highly endemic environment.	
